# Antistaphylococcal Activity and Phytochemical Analysis of Crude Extracts of Five Medicinal Plants Used in the Center of Morocco against Dermatitis

**DOI:** 10.1155/2019/1803102

**Published:** 2019-11-04

**Authors:** Ikrame Zeouk, Mounyr Balouiri, Khadija Bekhti

**Affiliations:** Department of Biology, Faculty of Sciences and Techniques, Sidi Mohamed Ben Abdellah University, Laboratory of Microbial Biotechnology, B.P. 2202 Imouzzer Road, Fez, Morocco

## Abstract

Novel drugs for methicillin-resistant *Staphylococcus aureus* (MRSA) hospital- and community-acquired infections are needed because of the emergence of resistance against antibiotics. In this study, methanolic and aqueous extracts of *Berberis hispanica*, *Crataegus oxyacantha*, *Cistus salviifolius*, *Ephedra altissima*, and *Lavandula dentata* selected from an ethnopharmacological study to treat skin infections in Sefrou city (Center of Morocco) were tested for their antistaphylococcal activity against strains often involved in cutaneous disorders: two methicillin-resistant *Staphylococcus aureus* strains and one strain of *Staphylococcus epidermidis* using the well-diffusion assay, while the agar macrodilution method was used to determine the minimal inhibitory concentrations. The total phenolic compounds and flavonoid contents of all tested extracts were also evaluated. Three of the five methanolic extracts showed an important antibacterial activity. *Berberis hispanica* extract was the most active with a minimal inhibitory concentration of 04.00 mg/ml against all tested strains, followed by *Cistus salviifolius* and *Crataegus oxyacantha* extracts containing the highest amounts of total phenols (133.83 ± 9.03 and 140.67 ± 3.17 *μ*g equivalent of gallic acid/mg of extract). However, the aqueous extracts have not shown any activity against the tested strains. The current data suggested that the most active extracts can be a good source of natural antistaphylococcal compounds and warrants further investigations to isolate bioactive molecules.

## 1. Introduction

The Gram-positive *Staphylococcus aureus* is a notorious pathogen responsible for diverse infections ranging from acute diseases such as skin abscesses, impetigo, and furunculosis [1, 2] to severe chronic infections [3, 4]. Due to multiple virulence factors, *S. aureus* can attach to host tissues and cause serious diseases. For example, toxigenic strains of this pathogen have been responsible for staphylococcal-scaled skin syndrome through the diffusion of the exfoliative toxins [5] and for necrotizing pneumonia through the secretion of Panton-Valentine leukocidin pore-forming toxin [6]. Among pathogenic *Staphylococcus* genus, *S. epidermidis* bacteria has also gained substantial interest since it is the most important cause of nosocomial infections. Contrary to *S. aureus*, *S. epidermidis* produces a limited number of toxins [7], and it was reported that only one toxin (the hemolytic peptide *δ*-toxin) has been involved in necrotizing enterocolitis in neonates [8]. It was suggested that the ability of *S. epidermidis* to adhere to surfaces and materials and to persist there declares it as a pathogen [7]. After the treatment failure of penicillins and semisynthetic penicillins, vancomycin has been the agent of choice to treat methicillin-resistant *Staphylococcus aureus* (MRSA) infections [9]. Unfortunately, multiresistance and toxicity of this antibiotic cannot be negligible [10, 11] and make the management of all those bacterial infections increasingly difficult which is a major prevalent worldwide cause of healthcare [12] and a significant cause of global morbidity and mortality [13]. This situation entails intensified efforts to develop alternative treatment. Natural substances could constitute a prominent source of new antibacterial molecules knowing that 75% of drugs against infectious diseases are natural products or natural derived products [14]. In the central north of Morocco, the geographical situation and the mild climate of Sefrou area make this city very rich in flora, sheltering various plants with medicinal properties. The most potent of these plants, *Berberis hispanica*, *Crataegus oxyacantha*, *Cistus salviifolius*, *Ephedra altissima*, *and Lavandula dentata* were subjected to the *in vitro* antistaphylococcal activity and phytochemical quantification. These plants are frequently used by local population to treat skin infections and known to possess several biological activities including antibacterial effect [15–18]. However, to best of our knowledge, *E. altissima* has never been reported for its antibacterial potent.

## 2. Materials and Methods

### 2.1. Plant Selection

The five studied plants were selected from an ethnopharmacological study undertaken in Sefrou city (Center of Morocco) [19]. Based on the calculated frequency index, the five plants were the most cited by herbalists to cure skin infections, and the used parts were also prescribed by herbalists (Table 1). Moreover, the studied plants are commonly known by the good antibacterial activities except for *E. altissima* species that has not been reported in the literature.

### 2.2. Plant Collection and Identification

Plants were purchased in February 2016 from the Atlas Mountains in the Imouzzer region (Morocco). Scientific names of species were identified by a specialist following “Morocco Flora” [20] and the endemic flora of Morocco [21]. Voucher specimens were deposited in the Laboratory of Microbial Biotechnology in the Faculty of Sciences and Techniques in Sidi Mohamed Ben Abdellah University of Fez, Morocco.

### 2.3. Extract Preparation

To obtain the crude extracts, two methods have been used: decoction to prepare aqueous extract and maceration to get the methanolic extract.

#### 2.3.1. Decoction

The decoction method described by Kengni et al. [22] has been used, the leaves of *C. oxyacantha*, *C. salviifolius*, and *L. dentata* and the roots of *E. altissima* and *B. hispanica* were grounded, 25 g of the powder of each plant was introduced in 250 ml of distilled water, and the mixture was boiled for 15 min, cooled, and filtered through Whatman n°1 paper and then evaporated under vacuum using a rotatory evaporator. The extracts were stored in a refrigerator at 4°C until further use.

#### 2.3.2. Maceration

The extraction method described previously [23, 24] has been used with a slight modification. Briefly, 25 g of the powder of each plant was macerated in 250 ml of methanol under agitation of 500 rpm at room temperature for 6 h. The resulting mixture was filtered using Whatman filter paper n°1 and then evaporated under vacuum. The residue obtained has been delipidated by hexane maceration (1 : 10 w/v) under agitation at room temperature for some minutes in order to eliminate lipids. Dried delipidated extracts were stored in a refrigerator at 4°C until further use.

### 2.4. Antibacterial Testing

#### 2.4.1. Target Microorganisms

The *in vitro* antistaphylococcal effect of both methanolic and aqueous extracts was tested against three strains of *Staphylococcus* including *S. aureus* ATCC 29213, *S. aureus* (clinical isolate), and *S. epidermidis* ATCC 12228, often involved in skin infections. These strains were maintained in 20% glycerol at 20°C as stock. The antibiogram profile of strains' bacteria was identified at the laboratory of bacteriology in the University Health Center-Fez, Morocco (Table 2).

#### 2.4.2. Inoculum Preparation

Revivification of bacteria has been performed by subculturing the agar plate surface Luria–Bertani (LB) prepoured in petri dishes and incubated at 37°C for 18 to 24 h. The microbial inoculums were obtained from fresh colonies using the direct colony suspension method. Hence, 1 to 2 colonies were suspended in a sterile saline (NaCl 0.9%) and compared with 0.5 McFarland standard to obtain standardized inoculums (10^8^ CFU/ml).

#### 2.4.3. Agar Well-Diffusion Method

As described by Balouiri et al. [25], the entire agar surface of the petri dish was inoculated spreading 1 ml of bacterial inoculums. The seeding of these inoculums was done to ensure a homogeneous distribution of bacteria. Aseptically, excess liquid was eliminated using a Pasteur pipette, and the plates were dried. After 30 min of the drying process in ambient temperature, a hole with a diameter of 6 mm was punched aseptically using a tip. The extract solution was filtrated, and then 80 *μ*l of each sterile stock (50 mg/ml: extract/distillate water) was introduced into each well. Finally, agar plates were incubated for 24 h at 37°C. Distillated water was used as negative control, while ampicillin (100 *μ*g/ml) was used as positive control. After measuring the diameter of inhibition zones around the well, the means were calculated. Tests were performed in triplicate.

A threshold was fixed: extracts with a diameter superior such as 10 mm were considered as active, and they were subjected to the determination of the minimum inhibitory concentration.

#### 2.4.4. Determination of the Minimum Inhibitory Concentration (MIC)

The MIC was determined using the agar dilution method as described by Balouiri et al. [25]. The method is based on the incorporation of varying concentrations of the extracts such as antimicrobial agent into the agar medium before its solidification. Different concentrations of each extract (ranging from 50 to 160 mg/ml per factor of 2) were prepared in dimethyl sulfoxide (DMSO) (20%), and 1 ml of each dilution was incorporated in 9 ml of sterile and soft LB as a medium culture. The mixture was grounded carefully and aseptically and then distributed into petri dishes. After the medium's solidification and from a suspension adjusted to 10^5^ UFC/ml, spots of 5 *μ*l were deposited aseptically on the agar surface. The dishes were incubated for 24 h at 37°C. A growth control was prepared without plant extracts.

#### 2.4.5. Target Strain Susceptibility Testing

The antibiogram profile of the target bacterial strains used in this study was evaluated against sixteen antibiotics belonging to various families including penicillins, cephalosporins, glycopeptides, macrolides, tetracyclines, polypeptides, as well as a penicillin combination (ampicillin/clavulanate) and other antibiotics (fusidic acid and pristinamycin).

The standardized disk-diffusion method was performed as described by the Clinical and Laboratory Standards Institute [26]. Briefly, inoculums have been prepared by a direct colony suspension (in sterile physiologic saline) from subcultures of the target strains on the Mueller–Hinton Agar (MHA) plates and then adjusted to the 0.5 McFarland scale. Sterile MHA plates were inoculated with the bacterial strains, and the commercial antibiotic disks were deposited aseptically on the agar surface. After incubation at 35°C for 16 to 18 h, the diameters of the inhibition zones were measured in mm and the strains were categorized according to the published standards [27].

#### 2.4.6. Total Phenolic Quantification

The total phenolic quantification was carried out using the Folin–Ciocalteu reagent by introducing 1.5 ml of this reagent (10%) in 200 *μ*l of extract (1 mg/ml methanol), and the mixture was agitated carefully and allowed to react for 5 min in dark, followed by addition of 1.5 ml of sodium carbonate (5%). After 2 hours of incubation in dark at room temperature, values were measured using a UV-visible spectrophotometer at 750 nm. Under the same conditions, a calibration range was made using gallic acid with different concentrations ranging between 300 *μ*g/ml and 25 *μ*g/ml. The total phenolic content was expressed as *μ*g gallic acid equivalents per mg dry weight of extract (eq GA/mg of E). The test was performed in triplicate.

#### 2.4.7. Total Flavonoid Quantification

The flavonoid content was determined as described by Bahorun et al. [28] 0.5 ml of each extract was mixed with 0.1 ml of aluminium chloride (10%), 0.1 ml of potassium acetate (1 M), and 4.3 ml of distillated water; after a vigorous agitation, the mixture was incubated for 30 min in ambient temperature. DO's values were measured using a UV-visible spectrophotometer at 415 nm. Flavonoid content was expressed as *μ*g quercetin equivalents per mg dry weight of extract (eq Que/mg of E) using a calibration range from 25 to 300 *μ*g/ml. The test was performed in triplicate.

### 2.5. Statistical Analysis

The results were presented as mean values ± Standard deviation (SD), and statistical analyzes were performed using ANOVA by IBM SPSS Statistics 21. Differences at *P* < 0.05 were considered statistically significant.

## 3. Results and Discussion

### 3.1. Antibacterial Activity Assay

The antibiogram of the clinical isolates and reference strains (Table 1) was determined at the laboratory of bacteriology UHC- Fez. According to the National Committee for Clinical Laboratory Standards (NCCLS), *S. epidermidis* is susceptible to all antibiotics, except tetracycline and colistin, while *S. aureus* ATCC 29213 and *S. aureus* (clinical isolate) are resistant to all tested antibiotics except vancomycin, teicoplanin, tetracycline, and pristinamycin. Both these *S. aureus* strains are methicillin resistant (MRSA). Indeed, MRSA bacteria have become a major health problem in both nosocomial and community-acquired infections [29, 30]. New effective antimicrobials are needed; natural products can be a source of potential antimicrobial agents against *S. aureus*, especially MRSA. Five medicinal plants (Table 2) were selected from an ethnopharmacological study in the Center of Morocco (data not shown). The data pertaining to the *in vitro* antistaphyloccocal activity of the crude extracts are presented in Tables 3 and 4. Based on these tables, the antibacterial activity is varied upon the plant extracts, the solvent, and the target microorganism. The aqueous extracts have not shown any inhibitory effect, while the methanolic extracts were active against all the tested strains. The methanolic extract of *B. hispanica* was the most active against all tested strains with a MIC of 4.00 mg/ml, followed by *C. oxyacantha* and *C. salviifolius* which is in accordance with the diameters of inhibition zones (ranging between 18.50 ± 0.70 and 12.50 ± 0.50 mm). The methanolic extracts of *E. altissima* roots and *L. dentata* leaves have shown a very weak activity, and the diameters of inhibition zones did not exceed 11.50 ± 0.50 mm. In addition to that, MRSA clinical isolate was more resistant to plant extracts than MRSA reference strain. The good activities of *B. hispanica*, *C. oxyacantha*, and *C. salviifolius* are in agreement with previous investigations. The antistaphylococcal activity of *B. hispanica* root extract is in accordance with a previous study, and it has been reported that the ethanolic extract of *B. hispanica* roots was active against *S. aureus* [15]. Mahmoudi et al. [31] have confirmed the antibacterial activity of the hydroalcoholic extract of *C. salviifolius* leaves against *S. aureus* ATCC 29213 with a MIC of 12.50 mg/ml. Moreover, Rebaya et al. [32] have shown that the ethanolic extract prepared from the arial part of *C. salviifolius* was active against various microorganisms, including *S. aureus*, and one of the leaves was more efficient against *S. aureus* (MIC = 1.562 mg/ml). This result shows that the used part is an important factor influencing the biological activity in addition to the nature of solvent, and Güvenç et al. [33] have confirmed that the extracts of *C. salviifolius* leaves and fruits exhibit varied antistaphylococcal activity with respect to the nature of solvents (water, methanol, chloroform, ethyl acetate, butanol, and remaining aqueous extract). This result confirms the obtained data: the methanolic extract of *C. salviifolius* was active, while the aqueous extract was not. Stelmakiene et al. [34] have shown that the aqueous extract prepared from the leaves of *C. oxyacantha* has revealed moderate antistaphylococcal activity against *S. aureus* and *S. epidermidis* (inhibition zones were 9 and 9.5 mm, respectively), while the compounds extracted from the leaves of *C. oxyacantha* were not active contrary to our results [35]. To the best of our knowledge, the antibacterial activity of neither the root extract of *E. altissima* nor the crude extract of *L. dentata* has been reported.

### 3.2. Quantitative Phytochemical Composition

The studied extracts were subjected to a phytochemical quantification of total phenols and flavonoids. The results of the preliminary phytochemical analysis have revealed the presence of phenols and flavonoids (Tables 5 and 6). From Table 5, (i) the highest concentration of total phenols was observed in *C. oxyacantha* and *C. salviifolius* extracts while *B. hispanica* water extract is the poorest; (ii) *L. dentata* methanolic extract has enclosed as much total phenols as water extract of *C. salviifolius*; however, the two extracts in this study do not exhibit antistaphylococcal activity (Table 3), and this result could suggest that the concentration of phenolic compounds may have an impact on the antimicrobial activity; (iii) methanolic extracts of all the tested plants were richer in phenolic compounds compared with water extracts except for *E. altissima* methanolic extract (*P* < 0.05). This result is probably owing to the nature and the polarity of solvents which influence the solubility of phytochemical compounds. For instance, previous studies have shown that the polarity of solvent has a significant impact on the extractability capacities of the phenolic compounds [36, 37]. For instance, methanol is less polar than water, but it is more efficient because it can release compounds easily from plant cell that have unipolar character [38]. Regarding flavonoids (Table 6), the aqueous and methanolic extracts of *C. salviifolius* were in the first rank, with an almost equal content followed by *L. dentata*, *C. oxyacantha*, and then the other extracts. It should be noted that the aqueous and the methanolic extracts of the five plants retain the same order in terms of flavonoid content except for *C. oxyacantha*.

Numerous causes may justify the variations of total phenolics and flavonoid amounts reported in this work. The variation of the polyphenolic content of a plant could be influenced by different biotic factors (plant species, used part, and physiological stage) and abiotic factors (environment and solvent) which could have an impact on the metabolism of plants [39]. Thus, (i) Rebaya et al. [32] have demonstrated that the extraction of the leaves of *C. salviifolius* using different solvents has an impact on the total phenols and total flavonoid contents and the aqueous extract has recorded the highest concentrations of total flavonoids and total phenols than the ethanolic extract [32]; (ii) Barrajón-Catalán et al. [40] were focused on the plant's organ, and they have demonstrated that *C. salviifolius* leaves contain more phytochemical compounds than the other organs; and (iii) Haouat et al. [41] have performed a phytochemical screening of the ethanolic extract of *B. hispanica* roots, and it was rich in flavonoids, total phenols, alkaloids, and tannins which in disagreement with our results. For *C. oxyacantha*, Stelmakiene et al. [34] have demonstrated that the aqueous extract prepared from the leaves was rich in phenolic compounds and the hydroethanolic extract was very rich of flavonoids.

The phytochemical compound mechanism of action as antimicrobial agents was proposed [17]. The methanolic extract prepared from the leaves of *C. salviifolius* is rich in total phenols and flavonoids, and these compounds exert an inhibitory activity against xanthine oxidase, acetylcholinesterase, and superoxide dismutase enzymes necessary for the bacterial metabolism. Various scientific investigations have explained the effectiveness of total phenols and flavonoids against pathogenic germs through a direct action or through the suppression of microbial virulence factors. For example, it was reported that flavonoids can inhibit some of bacterial virulence factors, including quorum-sensing signal receptors, enzymes, and toxins that are necessary for bacteria growth and metabolism [42]. The antibacterial activity of different groups of flavonoids can be attributable to other mechanisms such as the inhibition of energy metabolism of bacteria, the inhibition of nucleic acid synthesis, and the inhibition of cytoplasmic membrane function [43]. In the case of the genus *Staphylococcus*, it was demonstrated that flavonoids have an aggregatory effect on whole bacterial cells [44, 45]. Our results have also shown that *B. hispanica* is poor in total phenols and flavonoids, but it has shown the best antistaphylococcal activity and especially against MRSA which could be owed to another phytochemical family or specific active molecule. This hypothesis is confirmed by the bioguided fractionation assay reported by Aribi et al. [15], and they have isolated the most active fraction from *B. hispanica* Boiss. & Reut. against S. aureus and then identified the main compound as berberine tannate. This molecule synthesizes 5′-methoxyhydnocarpine-D (5′-MHC-D) and pheophorbide (chlorophyll decomposition products) that are responsible for the inhibition of efflux pump expression in *S. aureus* through the extrusion of antimicrobial agents from bacterial cells [46].

Since some of the studied plants in the current work are rich in flavonoids, an important point is to discuss the relationship between the antibacterial activity and the phytochemical compounds to better understand the mode of action of these entities. For example, the diversity of the flavonoid family makes it a prominent source of active molecules. In a recent review, the authors have summarized the proposed antibacterial mode of action of flavonoids and structure-activity relationship, especially for MRSA, and it was noted that the chalcones showed an interesting effect even stronger than the standard drugs [47]. Chalcones with a lipophilic group such as isoprenoid and methoxy groups at positions 3′, 5′, and 2′ of ring A are the most potent inhibitors of MRSA strains [48, 49]. Diverse mechanisms of action of flavonoid group were exposed; for instance, baicalein has been demonstrated to be able to reverse the ciprofloxacin resistance of MRSA through NorA efflux pump inhibitory effect, and also the inhibition of virulence factors of MRSA such as pyruvate kinase could lead to a deficiency of ATP [50]. However, a bioguided fractionation of the active species in the current study is needed even mandatory to identify the active molecules in order to be able to elucidate the mode of action and structure-activity relationship.

## 4. Conclusion

The methanolic extracts of *Berberis hispanica*, *Crataegus oxyacantha*, and *Cistus salviifolius* are the most active against *Staphylococcus* strains with MIC values ranging between 4.00 and 16.00 mg/ml. *Cistus salviifolius* and *Crataegus oxyacantha* extracts were very rich in total phenols. This evidence emphasizes the role of ethnopharmacological data as a framework for the discovery of bioactive compounds from plants. The antimicrobial profiles of *C. oxyacantha* and *C. salviifolius* leaf extracts may be explained by the high content of phytochemical families such as flavonoids and phenolic compounds, while berberine may be the active compound in *B. hispanica*. The active plants in the present work could be a good source for effective molecules used in drugs design against infectious diseases associated with *Staphylococcus* including MRSA infections. However, other complementary and deeper tests are recommended in order to purify and identify the specific active compounds.

## Figures and Tables

**Table 1:** The studied plants.

Plants (scientific name)	Family	Used parts
*Berberis hispanica* Boiss. & Reut.	Berberidaceae	Roots
*Crataegus oxyacantha* L.	Rosaceae	Leaves
*Cistus salviifolius* L.	Cistaceae	Leaves
*Ephedra altissima* Desf.	Ephedraceae	Roots
*Lavandula dentata* L.	Lamiaceae	Leaves

**Table 2:** Antibiogram profile of the target bacterial strains.

Antibiotic family	Antibiotic	Dose per disk (*μ*g)	*S. epidermidis*	*S. aureus* ATCC 29213
				*S. aureus* clinical isolate
Penicillins	Penicillin	10 units	Susceptible	Resistant
	Ampicillin	10	Susceptible	Resistant
	Amoxicillin	20	Susceptible	Resistant
	Oxacillin	1	Susceptible	Resistant
	Methicillin	5	Susceptible	Resistant
Penicillin combinations	Amoxicillin/Clavulanate	20/10	Susceptible	Resistant
Cephalosporins	Ceftriaxone	30	Susceptible	Resistant
	Ceftazidime	30	Susceptible	Resistant
Glycopeptides	Vancomycin	30	Susceptible	Susceptible
	Teicoplanin	30	Susceptible	Susceptible
Macrolides	Erythromycin	15	Susceptible	Resistant
	Spiramycin	15	Susceptible	Resistant
Tetracyclines	Tetracycline	30	Resistant	Susceptible
Polypeptides	Colistin	10	Resistant	Resistant
Others	Fusidic acid	10	Susceptible	Resistant
	Pristinamycin	10	Susceptible	Susceptible

**Table 3:** Antibacterial screening of plant extracts by the agar well-diffusion method.

Extract	*S. epidermidis*	*S. aureus*	*S. aureus* ATCC 29213
*Aqueous extracts*			
* B. hispanica*	—	—	—
* C. oxyacantha*	—	—	—
* C. salviifolius*	—	—	—
* E. altissima*	—	—	—
* L. dentata*	—	—	—
*Methanol extracts*			
* B. hispanica*	16.50 ± 0.50	12.50 ± 0.70	18.50 ± 0.70
* C. oxyacantha*	15.50 ± 0.50	12.50 ± 0.50	12.50 ± 0.70
* C. salviifolius*	13.50 ± 0.50	12.50 ± 0.50	13.00 ± 1.40
* E. altissima*	11.50 ± 0.50	10.50 ± 0.50	07.50 ± 0.50
* L. dentata*	10.50 ± 0.50	—	09.50 ± 0.50

Results are expressed as diameters of growth inhibition zones (mm) including the hole diameter (6.00 mm).

**Table 4:** Minimum inhibitory concentrations (mg/ml) of the most active studied extracts.

Target microorganisms	Methanol extracts		
	*B. hispanica*	*C. oxyacantha*	*C. salviifolius*
*S. epidermidis*	04.00	08.00	08.00
*S. aureus*	04.00	16.00	04.00
*S. aureus* ATCC 29213	04.00	08.00	08.00

**Table 5:** Total phenolic contents of the aqueous and methanol extracts.

Plant	Total phenolic contents (*μ*g equivalent of gallic acid/mg of extract)		*t*-test
	Methanol extracts	Aqueous extracts	
*C. salviifolius*	133.83 ± 9.03^a^	80.29 ± 4.16^a^	0.001
*C. oxyacantha*	140.67 ± 3.17^a^	51.96 ± 3.19^b^	0.000
*B. hispanica*	31.09 ± 1.80^b^	3.58 ± 0.38^d^	0.000
*L. dentata*	80.83 ± 4.91^c^	77.88 ± 1.60^a^	0.377
*E. altissima*	17.92 ± 1.25^b^	30.17 ± 1.09^c^	0.000

Means that not share the same letter per the same column are statistically different.

**Table 6:** Total flavonoid contents of the aqueous and methanol extracts.

Plants	Total flavonoid contents (*μ*g equivalent of quercetin/mg of extract)		*t*-test
	Methanol extracts	Aqueous extracts	
*C. salviifolius*	57.92 ± 2.46^a^	56.74 ± 0.48^a^	0.461
*C. oxyacantha*	34.80 ± 2.60^c^	15.56 ± 0.79^c^	0.000
*B. hispanica*	12.36 ± 0.84^e^	11.32 ± 1.39^d^	0.329
*L. dentata*	41.53 ± 1.74^b^	30.00 ± 0.21^b^	0.000
*E. altissima*	18.13 ± 1.05^d^	13.75 ± 1.81^c,d^	0.022

Means that not share the same letter per the same column are statistically different.

## Data Availability

Data used to support the findings of this study are included within the article and also available from the corresponding author upon request.
